# Dual-Stage Clean-Sample Selection for Incremental Noisy Label Learning

**DOI:** 10.3390/bioengineering12070743

**Published:** 2025-07-08

**Authors:** Jianyang Li, Xin Ma, Yonghong Shi

**Affiliations:** 1 Academy of Engineering & Technology, Fudan University, Shanghai 200433, China; jianyangli20@fudan.edu.cn; 2 Digital Medical Research Center, School of Basic Medical Science, Fudan University, Shanghai 200032, China; 3 Shanghai Key Laboratory of Medical Image Computing and Computer Assisted Intervention, Shanghai 200032, China; 4 Shanghai Sixth People’s Hospital Affiliated to Shanghai Jiaotong University, Shanghai 200233, China

**Keywords:** class-incremental learning, noisy label, image classification, memory rehearsal

## Abstract

Class-incremental learning (CIL) in deep neural networks is affected by *catastrophic forgetting* (CF), where acquiring knowledge of new classes leads to the significant degradation of previously learned representations. This challenge is particularly severe in medical image analysis, where costly, expertise-dependent annotations frequently contain pervasive and hard-to-detect noisy labels that substantially compromise model performance. While existing approaches have predominantly addressed CF and noisy labels as separate problems, their combined effects remain largely unexplored. To address this critical gap, this paper presents a dual-stage clean-sample selection method for Incremental Noisy Label Learning (DSCNL). Our approach comprises two key components: (1) a dual-stage clean-sample selection module that identifies and leverages high-confidence samples to guide the learning of reliable representations while mitigating noise propagation during training, and (2) an experience soft-replay strategy for memory rehearsal to improve the model’s robustness and generalization in the presence of historical noisy labels. This integrated framework effectively suppresses the adverse influence of noisy labels while simultaneously alleviating catastrophic forgetting. Extensive evaluations on public medical image datasets demonstrate that DSCNL consistently outperforms state-of-the-art CIL methods across diverse classification tasks. The proposed method boosts the average accuracy by 55% and 31% compared with baseline methods on datasets with different noise levels, and achieves an average noise reduction rate of 73% under original noise conditions, highlighting its effectiveness and applicability in real-world medical imaging scenarios.

## 1. Introduction

Recent advances in deep learning have revolutionized medical image analysis [1], enabling breakthroughs in lesion detection [2], segmentation [3], and classification [4]. However, the dynamic nature of clinical data—driven by evolving disease subtypes, imaging modalities, and diagnostic standards—challenges the traditional independent and identically distributed (i.i.d.) data assumption [5]. Conventional deep neural networks struggle with this reality: full retraining for new data or tasks is computationally prohibitive, while incremental updates risk catastrophic forgetting, where newly acquired knowledge significantly degrades prior learning [6]. Addressing these limitations, such as enabling models to efficiently assimilate new information while preserving existing knowledge, is a critical challenge in adapting deep learning to real-world clinical evolution.

Incremental learning has emerged as a key solution to catastrophic forgetting, aiming to enable models to acquire new tasks or classes while retaining previously learned knowledge without requiring access to the entire historical dataset [7]. Current approaches primarily include regularization-based, rehearsal-based, optimization-based, feature-decoupling, and architecture-expansion methods, with rehearsal-based strategies being particularly prevalent due to their implementation simplicity, stable performance, and multi-task adaptability [7]. These methods mitigate forgetting by storing or regenerating previous data, such as past samples, feature representations, or training gradients, and jointly training them with new data [8,9]. However, when applied to medical imaging, these techniques face unique challenges, including domain-specific complexity due to distinctive anatomical structures and pathological patterns, data scarcity constraints that limit rehearsal effectiveness, and the risk of amplifying noisy labels during incremental updates. These unresolved issues underscore the need for specialized incremental learning frameworks tailored to the distinctive requirements of medical image analysis.

**Figure 1:**
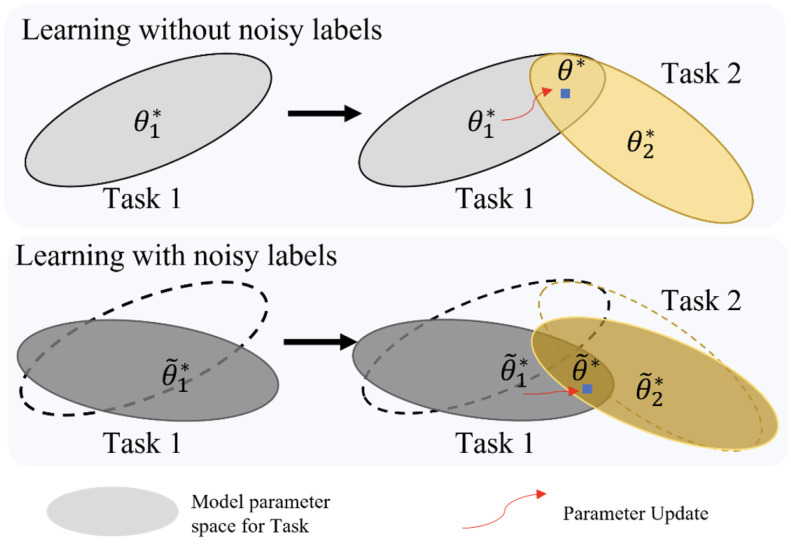
Noisy labels disrupt parameter constraints, exacerbating catastrophic forgetting in incremental learning. (**Top**) Conventional incremental learning mitigates forgetting by constraining parameters within flat loss regions across tasks. (**Bottom**) constraint regions with biased gradients. When subsequent incremental learning attempts to optimize based on these noise-contaminated parameter spaces, the accumulated deviations significantly intensify catastrophic forgetting.

Particularly, medical image annotation presents unique challenges due to its costly, expertise-driven nature and inherent inter-annotator variability stemming from ambiguous semantic boundaries. As a result, noisy labels are pervasive, especially when new classes are continuously introduced, posing elevated demands on the noise robustness of incremental learning models [10]. Critically, the replay mechanism widely used in incremental learning is built upon a key assumption that the stored historical labels are perfectly accurate. However, this assumption rarely holds in real-world medical settings and is often overlooked. When mislabeled samples are repeatedly replayed during incremental training, the adverse effects of noisy labels are amplified, leading to a compounding replay error that undermines both the learning of new tasks and the retention of previous knowledge.As illustrated in Figure 1, noisy labels disrupt parameter constraints in incremental learning, thereby exacerbating catastrophic forgetting.

The optimization of models in incremental scenarios complicated by noisy labels is defined as Incremental Noisy Label Learning. This challenge inherently comprises two intertwined problem settings: the incremental learning paradigm and the learning with noisy labels paradigm. Within this research problem, the incremental learning scenario can be considered the foundational underlying problem, while the presence of noisy labels acts as a critical, real-world constraint. Existing research primarily explores two directions individually to address this joint challenge. One line of work focuses on enhancing model stability during the learning process by mitigating catastrophic forgetting through techniques such as gradient projection or constrained optimization, as exemplified by methods like GEM [11] and A-GEM [12]. The other line centers on improving robustness to noisy labels using strategies such as loss reweighting [13], sample selection [14], or consistency regularization [15]. While these individual advancements are significant, they fall short of fully addressing the complex Incremental Noisy Label Problem. These approaches are generally designed in isolation, with limited adaptability to settings where both challenges coexist. This limitation is particularly critical in medical imaging scenarios, where continual class expansion and pervasive noisy labels are frequently intertwined. Currently, there is a lack of a unified robust learning framework capable of jointly modeling and addressing both catastrophic forgetting and label noise. This gap significantly hinders the reliable application of incremental learning techniques in real-world clinical environments, making the Incremental Noisy Label Problem a crucial and urgent area for research.

To address the complex challenge of coexisting incremental class and label noise in medical image classification, this study proposes a novel framework termed dual-stage clean-sample selection for Incremental Noisy Label Learning (DSCNL). Built upon a replay-based architecture, DSCNL introduces robust learning modules at two critical stages, such as initial task training and replay-sample selection, to systematically enhance the model’s noise resistance and replay stability throughout the incremental learning process. Specifically, during the initial training of each task, a filtering module dynamically evaluates label reliability based on prediction confidence from an auxiliary model and historical probability confidence distributions. This enables the construction of a “clean” training subset that reduces the interference of noisy samples with feature learning. Meanwhile, replay-sample selection is optimized via representativeness and diversity-aware sampling to enhance knowledge retention. In the subsequent incremental phases, an experience soft-replay strategy, utilizing image mixing and soft label reconstruction, counteracts the accumulation of noisy labels in replayed data, thereby suppressing the propagation of erroneous knowledge and improving the model’s robustness. This dual-stage denoising approach uniquely implements the hierarchical suppression of noise across both training and replay pathways. Extensive experiments on multiple medical image incremental classification benchmarks, under varying class incrementation scenarios and noisy label levels, demonstrate that DSCNL consistently outperforms representative incremental learning baselines across a range of evaluation metrics. Its remarkable stability under high noise and significant task-transfer settings underscores its potential for reliable deployment in real-world medical environments.

The main contributions of this study are summarized as follows:

- The combined impact of dynamic class incrementation and noisy labels in medical image classification was systematically analyzed, highlighting a significant oversight in existing incremental learning research when addressing this compounded challenge.
- A unified deep learning framework is proposed, integrating a clean-sample filtering mechanism during initial task training with an experience soft-replay strategy during replay. This design effectively mitigates both catastrophic forgetting and the negative impact of noisy labels, thereby significantly enhancing training stability and model robustness in incremental learning.
- A replay-sample selection strategy based on dynamic diversity estimation is introduced, which effectively balances sample representativeness and diversity. This approach leads to a more efficient memory buffer that better supports incremental learning.
- Extensive experiments are conducted on multiple public medical imaging datasets under various incremental classification settings. The results show that our method consistently outperforms mainstream incremental learning approaches across key evaluation metrics, demonstrating superior robustness and generalization, particularly under challenging conditions such as high noise rates and multi-phase class expansion.

## 2. Related Studies

### 2.1. Incremental Learning

Incremental learning, also known as continual learning, is a critical paradigm that enables models to continuously learn new classes from a stream of tasks while mitigating the catastrophic forgetting of previously learned information. This capability is vital for real-world applications where data arrives sequentially and models need to adapt without complete retraining. With the ongoing research in the field of incremental learning, five main research branches have emerged.

The first category comprises regularization-based methods. These approaches incorporate penalty terms into the objective function to safeguard previously learned knowledge. LwF (Learning without Forgetting) [16], for instance, uses knowledge distillation to constrain parameter updates based on differences between new and old tasks. LwM (Learning without Memorizing) [17] leverages unlabeled external data to combat forgetting, while EBLL (Elastic Boundary Lifelong Learning) [18] employs an auto-encoder to capture task-specific features. EWC (Elastic Weight Consolidation) [19] identifies important weight parameters using the Fisher information matrix. However, EWC can become overly restrictive with many tasks, hindering the learning of new information. To address this, methods like SI (Synaptic Intelligence) [20] determine parameter importance based on their variation range, and MAS (Memory-Aware Synapses) [21] allows for unsupervised importance weight estimation.

The second category consists of rehearsal-based methods. These strategies store a small subset of old data or generate synthetic samples from past tasks, then “rehearse” them alongside new data. This direct replay helps prevent the model from overwriting old knowledge. A key challenge lies in selecting appropriate old task samples to maximize the reuse of prior knowledge, given limitations in storage and computational resources. Some works employ random sample selection [22], while others impose further constraints to select representative [23] or diverse samples [24]. More advanced methods based on gradients, such as GEM (Gradient Episodic Memory) [11] and A-GEM (Averaged Gradient Episodic Memory) [12], construct constraints based on old samples to ensure their losses do not increase.

The third category includes optimization-based methods. These approaches constrain the optimization direction of weights in the parameter space to achieve a balance between plasticity (learning new tasks) and stability (retaining old knowledge). OWM (Orthogonal Weight Modification) [25] and AOP (Adaptive Orthogonal Projection) [26] update weights along directions orthogonal to previous tasks. Similarly, [27] utilizes orthogonal low-rank vector subspaces, and GPM (Gradient Projection Memory) [28] preserves the gradient subspace importance for past tasks through orthographic projection.

The forth category includes feature-decoupling methods. They aim to separate task-specific or class-specific features from more general, shared representations to mitigate interference between tasks. This often involves learning disentangled representations where certain parts of the feature space are dedicated to new knowledge without corrupting existing knowledge. While not explicitly detailed in the current text under a dedicated heading, some architecture-based or meta-learning approaches might implicitly leverage feature decoupling by activating different parts of the network or learning task-specific embeddings. For instance, HAT (Hard Attention to the Task) [29], by masking previous task parameters, effectively creates a form of feature decoupling by isolating task-specific pathways. Further exploration in this domain focuses on ensuring that features learned for new classes do not overlap detrimentally with those of old classes, potentially through methods that promote sparsity or orthogonality in feature representations across tasks.

The fifth category encompasses architecture-based methods, which adapt the network’s structure to accommodate new information. This can involve activating different parameters for distinct tasks or adding new parameters for incoming tasks. PackNet [30] prunes weights based on importance, training only a subset for the current task. Alternatively, some methods dynamically increase network structure by adding neurons for new tasks, as seen in Progressive Networks [31], Dynamic Memory Networks [32], and DER [33]. While many of these approaches require a task identifier, limiting them to multi-head setups, methods like Expert Gate [34] use auto-encoder gates to avoid this issue. However, increasing the network size can pose parameter limitations for large-scale tasks.

Despite significant research advancements in continual learning, a prevailing assumption across many existing methods is the absence of noisy labels within the datasets. This oversight presents a critical vulnerability: the effectiveness of both gradient-based and constraint-based methods is severely hampered by the negative interference of label noise. Such noise can corrupt the learning process, leading to suboptimal parameter updates and the inaccurate preservation of knowledge. Research specifically addressing the coexistence of continual learning and noisy labels remains relatively scarce, highlighting a crucial gap in the current literature.

### 2.2. Learning with Noisy Labels

Learning with noisy labels is a critical area of research that aims to mitigate the detrimental effects of incorrect labels on model training. The primary goal is to prevent the model from overfitting to noise, which can severely degrade its generalization ability. Researchers have developed several key strategies to address this challenge.

One prominent strategy focuses on sample selection and reweighting. These methods either identify and utilize purportedly clean samples for training or assign different weights to samples based on their likelihood of being corrupted. For instance, some approaches might train multiple networks that cross-check each other’s predictions to identify small-loss samples for training [14,35], while others use statistical measures like Area Under the Margin (AUM) to differentiate clean data from mislabeled instances, effectively discarding the latter by assigning them zero weight [36].

Another significant direction is label correction. This involves actively modifying or refining the noisy labels themselves during the training process. This can range from iteratively optimizing network parameters and labels to progressively correcting labels based on model confidence [37] or updating label distributions in an end-to-end manner [38]. The core idea is to transform the unreliable initial labels into more accurate ones that guide the learning process effectively.

Finally, robust loss functions are designed to make the training process inherently more tolerant to noise without directly altering samples or labels. These methods often involve either designing novel loss functions that are less sensitive to mislabeled examples (e.g., mean absolute error [39]) or dynamically adjusting the contribution of individual samples to the loss. This can be achieved through mechanisms like estimating noise transition matrices to correct the loss function [40] or employing techniques that aim to eliminate the influence of noisy samples on the optimization landscape [41].

## 3. Methods

### 3.1. Problem Settings

This study focuses on the class-incremental learning (CIL) problem [42], modeling it as the process of learning from a sequence of tasks, indexed by t=1,2,⋯,T. For each task *t*, given a dataset $D_{t}={\left\{\left(x_{i},y_{i}\right)\right\}}_{i=1}^{N_{t}}$ consisting of $N_{t}$ input samples $x_{i}$ and their corresponding labels $y_{i}$, these samples are drawn from an underlying experience distribution $D_{t}$ that follows the independent and identically distributed (i.i.d.) assumption. Let $K_{t}$ denote the set of unique class labels introduced in task *t*. The experimental design adheres to the standard CIL setup, where the class sets of different tasks are disjointed $K_{i}\cap K_{j}=⌀$ for i≠j. After learning task *t*, the model $f_{\theta }$ parameterized by θ is able to accurately classify samples from all classes encountered so far, i.e., classes in ${⋃}_{c=1}^{t}K_{c}$. The ideal objective is to minimize the expected loss function over all seen tasks to compute the optimal network parameters $\theta^{*}$, as shown in Equation (1):

(1) $$\theta^{*}=\underset{\theta }{\mathrm{argmin}}E_{t∼T}\left[E_{D_{t}}\left[L\left(\theta ;D_{t}\right)\right]\right]=\underset{\theta }{\mathrm{argmin}}E_{t∼T}\left[E_{\left(x_{i},y_{i}\right)∼D_{t}}\left[L\left(f_{\theta }\left(x_{i}\right),y_{i}\right)\right]\right]$$

where *L* is the training loss function, and *E* denotes the expectation.

To alleviate the problem of catastrophic forgetting caused by the continuous changes in the objective function due to task shifts in class-incremental learning, this study employs a fixed-size memory buffer $M={\left\{\left(x_{r},y_{r}\right)\right\}}_{r=1}^{N_{M}}$ to store and replay historical sample pairs sampled from the training data stream. Incremental learning methods based on historical sample replay typically optimize an objective function, as shown in Equation (2):

(2) $$\theta^{*}=\underset{\theta }{\mathrm{argmin}}E_{t∼T}\left[E_{D_{t}}\left[L\left(\theta ;D_{t}\right)\right]+E_{M}\left[L_{R}\left(\theta ;M\right)\right]\right]$$

where $L_{R}$ represents the sample replay loss function value generated by different replay-based methods. The ultimate convergence of this objective function is achieved when the minimal loss is obtained by $f_{\theta }$ for any given task *t*. This study adopts the classic experience replay method [43], where the replay loss function is defined as shown in Equation (3):

(3) $$L_{R}=E_{(x_{r},y_{r})∼M}\left[L\left(f_{\theta }\left(x_{r}\right),y_{r}\right)\right]$$

Previous CIL studies have seldom considered noisy labels. In this study, the possibility of incorrect annotations ${\tilde{y}}_{i}$ for samples $x_{i}$ from noisy dataset ${\tilde{D}}_{t}={\left\{\left(x_{i},{\tilde{y}}_{i}\right)\right\}}_{i=1}^{N_{t}}$ in task *t* is accounted for within real-world scenarios. In this case, the objective function is modified as shown in Equation (4):

(4) $$\theta^{*}=\underset{\theta }{\mathrm{argmin}}E_{t∼T}\left[E_{\left(x_{i},{\tilde{y}}_{i}\right)∼D_{t}}\left[L\left(f_{\theta }\left(x_{i}\right),{\tilde{y}}_{i}\right)\right]+E_{(x_{r},{\tilde{y}}_{r})∼M}\left[L\left(f_{\theta }\left(x_{r}\right),{\tilde{y}}_{r}\right)\right]\right]$$

where ${\tilde{y}}_{r}$ represents the potentially noisy labels of the historical samples stored in the memory.

As shown in Equation (4), label noise simultaneously impacts both the learning of new tasks during the initial training phase and the knowledge consolidation of old tasks during the replay enhancement phase. This leads to the accumulation of noise bias, thereby weakening the ability of rehearsal-based methods to mitigate catastrophic forgetting. Therefore, this study proposes a framework comprising three distinct modules, designed to selectively filter clean-labeled samples at multiple levels while enhancing sample diversity during the replay phase to address the label noise issue in class-incremental learning.

### 3.2. Overview of Framework

This study aims to effectively mitigate the performance degradation and catastrophic forgetting caused by noisy labels by implementing a dual clean-labeled sample filtering mechanism during both the initial training phase and the subsequent sample replay phase. Preliminary experiments show that directly training the model without addressing noisy labels leads to a significant drop in performance on the validation set. More critically, the sample (experience) replay buffer constructed under such training conditions inevitably contains a large number of incorrectly labeled image samples. This accumulation of noise results in a significant shift in the parameter space when the model later uses the replayed samples to consolidate previously learned knowledge, potentially causing a sharp collapse in model performance.

Based on the above observations, this study proposes the hypothesis that if clean-labeled samples can be effectively selected during the initial training phase of the model on current task data, and if sample purity and replay value are considered during the replay sample selection phase, along with robust training on potentially noisy historical samples during the replay-enhancement phase, it will collaboratively mitigate performance degradation during both the current task’s training and the knowledge retention process (replay phase). The final experimental results confirm the validity of this hypothesis, demonstrating that the proposed dual-filtering strategy plays a positive role in addressing the label noise problem in class-incremental learning.

**Figure 2:**
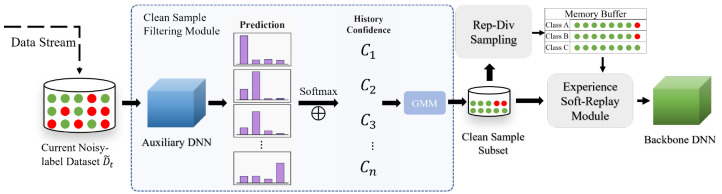
The overall framework of DSCNL. Training data with noisy labels are filtered through the clean-sample filtering module, selecting a high-confidence clean-sample subset for training the incremental backbone model. The representative-diversity (Rep-Div) sampling module selects the most valuable samples from the clean-sample subset and stores them in the memory buffer. During replay training, mixed samples with soft labels are synthesized using the experience soft-replay module, enhancing the model’s robustness.

To achieve clean-sample selection during both the training and replay phases, this study proposes a class-incremental learning model based on a dual-stage clean-sample filtering mechanism, as shown in Figure 2. The entire framework consists of three core modules, each designed to progressively enhance the purity of samples used for model training at different stages.

1. Clean-Sample Filtering Module for Initial Training: This module intervenes during the initial learning phase of the model on the new task data. By introducing an auxiliary expert network, it evaluates and filters the quality of the training samples for the current task. The core objective is to construct a high-confidence clean-sample subset, ensuring that the model can train on reliable label information during the early stage, thus minimizing the initial impact of label noise.
2. Representative-Diversity Sample Selection Module: When constructing the memory buffer for experience replay, this module is responsible for sampling from the historical task samples. Rather than selecting samples randomly, it evaluates candidate samples based on their representativeness (i.e., how well the sample reflects typical characteristics of its class) and diversity (i.e., the information variance between samples). The goal is to select those samples that most effectively support the model’s generalization and aid in retaining old knowledge. This strategy is designed to improve replay training efficiency and better mitigate catastrophic forgetting.
3. Experience Soft-Replay Module: This module comes into play when the model uses replay samples for knowledge consolidation. It merges current task samples with replay samples extracted from the memory buffer for joint training. A key feature of this module is the introduction of a robust learning mechanism based on soft labels, which aims to reduce the negative impact of potential incorrect labels in the replay samples on the incremental training process, enabling the soft replay of old knowledge.

By applying multi-level filtering and dynamic enhancement of sample quality in both the initial training and experience replay phases, the proposed method significantly alleviates model performance degradation and knowledge forgetting caused by noisy labels, and the data distribution shifts accompanying task sequences. This ultimately enhances the overall robustness and generalization ability of models trained in class-incremental learning scenarios. The specific implementation of each module will be detailed in the following sections.

#### 3.2.1. Clean-Sample Filtering Module

The core objective of the incremental learning model proposed in this study is to establish a replay storage mechanism with high purity, aiming to minimize the negative impact of potential historical labeling errors on the model’s overall performance during subsequent experience replay. To achieve this, before samples are selected for inclusion in the replay buffer, the model (or associated mechanisms) must be able to effectively distinguish between clean-labeled samples and noisy-labeled samples, ensuring that only the former are sampled for replay. However, preliminary experiments reveal significant challenges when relying on the same model to simultaneously perform both new knowledge learning and sample quality filtering. This challenge primarily arises from the old knowledge acquired by the model in previous tasks, which may interfere with the sample selection process for the current task, leading to confusion in the filtering results and ultimately affecting the accurate identification of clean samples.

To mitigate and overcome this inherent conflict, this study proposes a self-filtering sample module that introduces an independent auxiliary expert network, specifically designed to assess and filter the quality of input samples during the initial learning phase of the current task *t*.

**Figure 3:**
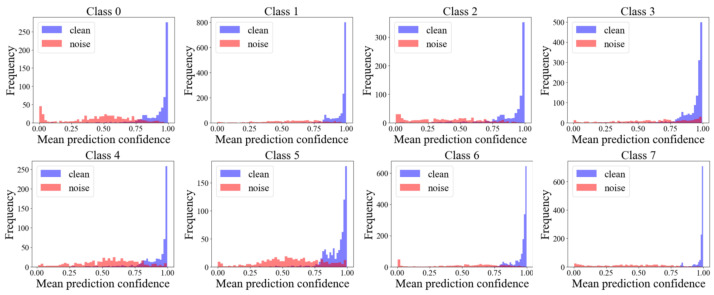
The distribution of historical average confidence on the labeled class after the warming-up phase. Red represents noisy-labeled samples, while blue represents clean-labeled samples.

In noisy label robust learning, a common strategy is to filter high-confidence samples based on metrics such as loss values or prediction confidence. Given that neural networks tend to first fit simple and correctly labeled samples (manifested as low loss) during the early stages of training, gradually learning and memorizing noisy labels (which typically have higher initial loss, leading to eventual overfitting and decreased generalization), existing methods like Co-teaching leverage this “low-loss” characteristic. However, single-pass loss and prediction probability (the latter being more interpretable) are susceptible to training fluctuations. To overcome this instability, this study proposes using the average historical prediction probabilities accumulated by the model during the early training phase as a more stable criterion for clean-sample selection. Our experimental observations (as shown in Figure 3) clearly indicate that clean samples generally exhibit significantly higher average historical prediction probabilities for their labeled classes, whereas noisy samples show the opposite pattern. Based on this distributional difference, an effective threshold can be set to separate the high-confidence “clean” sample subset ${\bar{D}}_{t}$ from the original training data ${\tilde{D}}_{t}$.

This study proposes a noise sample selection method based on the average historical prediction probability within each class, modeling the distribution of this metric for samples within a single class subset using a Binary Gaussian Mixture Model (BGMM). Compared to modeling the loss distribution across the entire training batch, this method allows for more precise differentiation between clean- and noisy-labeled samples. The core mechanism and process of this module are described as follows:

(1) Data and Historical Probability Storage: During the early stages of training, the historical buffer H records and stores the predicted probability of each sample belonging to its class subset for its labeled class on a per-iteration basis, as history confidence:

(5) $$H=\{C_{i}|C_{i}=\sum p\left(y={\tilde{y}}_{i}|x_{i}\right),i\in N_{t}\}$$

where $p(y={\tilde{y}}_{i}|x_{i})$ refers to the predicted probability value after softmax of the sample $x_{i}$ for its corresponding label ${\tilde{y}}_{i}$, as determined by the auxiliary model. $N_{t}$ is the total number of samples in ${\tilde{D}}_{t}$. After each training epoch, $C_{i}$ will be updated by summing it with the probability value.

(2) Dynamic BGMM Modeling and Observation: After each training iteration, the average historical prediction probabilities of the samples within each class subset are fitted using the BGMM. It is observed that, as training progresses, the mean centers of the two Gaussian components fitted by the BGMM (representing clean samples and noisy/difficult samples, respectively) undergo a dynamic change of “approach-separation-approach”. The “separation” phase provides the optimal opportunity for differentiation. The component with the larger mean indicates easily predictable clean/easy-to-learn samples, while the component with the smaller mean indicates difficult-to-predict noisy/hard-to-learn samples.

(3) Dynamic Threshold Selection and Auxiliary Network Training: Based on the BGMM, the probability of each sample belonging to the clean- or noisy-sample distribution is calculated. A dynamic amplification threshold processor is then used to select high-confidence clean samples. These selected clean samples are specifically used for training the auxiliary network.

(4) Iterative Optimization and Stopping Criteria: The auxiliary network is trained only on the currently selected clean samples, and its predictions for all samples are used for the next round of BGMM re-estimation and updating the clean-sample set ${\bar{D}}_{t}$. This iterative process continues until the number of clean samples selected by the auxiliary network reaches the expected total number of clean samples, which is determined based on the dataset’s inherent noise rate estimated through cross-validation. Once this condition is met, the training stops, and the final clean data subset ${\bar{D}}_{t}={\left\{\left(x_{i},{\bar{y}}_{i}\right)\right\}}_{i=1}^{{\bar{N}}_{t}}$ is obtained, where ${\bar{N}}_{t}$ is the total number of samples in the clean subset ${\bar{D}}_{t}$, and $x_{i}$ and ${\bar{y}}_{i}$ represent the selected sample and its clean label in the current task dataset.

#### 3.2.2. Representative–Diverse Memory Sampling

Upon completion of clean-sample selection, the Representative–Diverse Memory Sampling strategy is introduced, aimed at optimizing the replay sample selection in incremental learning. This method balances sample representativeness (typically close to the class center, where model predictions are stable) and diversity (possibly near the class boundary or feature-sparse regions, where model prediction uncertainty is higher). By addressing the limitations of traditional sampling methods (e.g., reservoir sampling, random sampling) in balancing both factors, this strategy enhances the model’s generalization ability in incremental multi-task learning.

The core mechanism of this method lies in the trade-off between representativeness and diversity, as well as the efficient estimation of uncertainty based on data augmentation. For the former, we assume that samples close to the class center are highly representative, while those near the class boundary exhibit strong discriminability, contributing to diversity. The sampling strategy aims to capture both types of samples, constructing a replay set that not only consolidates old knowledge but also effectively supports learning new tasks.

For the latter, efficient uncertainty estimation based on data augmentation, the motivation is to avoid the high computational cost of estimating the relative position of samples in the unified feature space. Model prediction uncertainty is utilized as an effective proxy for the sample’s position in the feature space. The core assumption is that the model has lower uncertainty when predicting samples closer to the class distribution center. Specifically, inspired by [44], data augmentation is leveraged to indirectly quantify the uncertainty of samples. For each original sample $x_{i}$, S stochastically augmented versions are generated, denoted as ${\hat{x}}_{i}^{1},{\hat{x}}_{i}^{2},\ldots ,{\hat{x}}_{i}^{S}$. These augmented samples are created by applying randomly selected data augmentation techniques (such as color transformation, intensity transformation, spatial transformation, and mix-type transformation) to $x_{i}$.

These augmented samples share the same label as the original sample $x_{i}$. The model then makes predictions on these *S* augmented samples. The predictive probability distribution for the original sample $x_{i}$ is approximated by averaging the predictions from its augmented versions, which is a form of Monte Carlo estimation, shown as Equation (6):

(6) $$p\left(y=c|x_{i}\right)=\frac{1}{S}\sum_{j=1}^{S}p\left(y=c|{\hat{x}}_{i}^{j}\right)$$

Here, $p(y=c|x_{i})$ represents the approximated posterior probability that sample $x_{i}$ belongs to class *c*, ${\hat{x}}_{i}^{j}$ is the *j*-th augmented version of $x_{i}$, and *S* is the total number of augmented versions generated for Monte Carlo sampling (other measures, like the entropy or variance of the averaged probabilities, can also be used). Each augmented sample ${\hat{x}}_{i}^{j}$ is generated by the augmentation function $f_{s}$ (with parameter $\theta_{s}$), randomly selected from a set of *S* available augmentation methods:

(7) $${\hat{x}}_{i}^{j}=f_{s}\left(x_{i}|\theta_{s}\right)$$

where *s* is randomly chosen from set {1,2,…,S} for each *j*. The implicit prior probability $p({\hat{x}}_{i}^{j}|x_{i})$ simply reflects this stochastic augmentation process, which can be defined as

(8) $$p\left({\hat{x}}_{i}^{j}|x_{i}\right)=\sum_{s=1}^{S}\omega_{s}*f_{s}\left(x_{i}|\theta_{s}\right)$$

where $\omega_{s}$ is a random one-hot encoding used to determine which augmentation method is chosen.

Sample uncertainty is then evaluated based on the consistency of predictions across these *S* augmented versions. If the model’s predictions for all augmented versions ${\hat{x}}_{i}^{j}$ are highly consistent, the uncertainty is considered low (indicating strong representativeness). One way to quantify this is by looking at the dispersion of prediction. A “vote count” $V_{c}$ for each class c based on the most likely predicted class for each augmented sample can be calculated:

(9) $$V_{c}\left(x_{i}\right)=\sum_{j=1}^{S}1_{c}\left(c=\underset{y}{\arg\max}\;p\left(y|{\hat{x}}_{i}^{j}\right)\right)$$

where 1 is an indicator function (1 if true, 0 if false) used to determine whether the predicted class matches the corresponding label.

Then the uncertainty $U_{x}$ of the sample $x_{i}$ is defined as 1 minus the proportion of votes for the most frequently predicted class (the mode of the empirical distribution):

(10) $$U_{x}\left(x_{i}\right)=1-\frac{1}{S}\max_{c}V_{c}\left(x_{i}\right)$$

The value of sample uncertainty decreases as the model’s predictions align more consistently with the labeled class *c*. A lower uncertainty value indicates more stability in predictions, suggesting that sample $x_{i}$ lies within the high-confidence region of the model’s sample space.

To maintain class balance, this study constructs the clean-sample replay memory buffer $M={\left\{\left(x_{r},{\bar{y}}_{r}\right)\right\}}_{r=1}^{N_{M}}$ of total size $N_{M}$. Specifically, the study allocates an equal number of memory blocks to each class in the trained model. For the clean-sample subset ${\bar{D}}_{t}$ of the current task *t*, a sampling strategy that balances both diversity and representativeness is followed. First, the uncertainty for each sample is computed, and the samples are then sorted based on their uncertainty values. Next, memory quotas are adjusted and diversity sampling is performed. The number of memory samples allocated to each class is redistributed based on predefined interval sizes as $\frac{N_{M}}{|{⋃}_{c=1}^{t}K_{c}|}$, where $|{⋃}_{c=1}^{t}K_{c}|$ is the total number of learned classes. For the new sample space, samples from the sorted list according to the sampling intervals ${\bar{D}}_{t}*\frac{|{⋃}_{c=1}^{t}K_{c}|}{N_{M}}$ are selected, ensuring that samples are drawn from the entire uncertainty spectrum (ranging from low-uncertainty robust samples to high-uncertainty fragile samples). This sampling strategy aims to systematically cover samples across different levels of uncertainty, ensuring both the diversity and uncertainty of the samples in the replay memory buffer. However, it is important to note that since this method aims to capture a broad range of samples, including “fragile” (high-uncertainty) ones, it may inadvertently introduce latent noisy-labeled samples into the memory buffer that were not completely removed during the initial clean-sample subset selection phase.

#### 3.2.3. Experience Soft-Replay Module

Following the prerequisite stages of clean-sample selection and representative-diverse replay sampling, the replay training phase is pivotal. It aims to optimize the fusion of new and old task data while overcoming potential noisy labels in historical samples. This phase employs the experience soft-replay module, where the training batch *B* consists of the current clean-sample subset ${\bar{D}}_{t}$ and samples drawn from the memory buffer *M*, as $B∼({\bar{D}}_{t}\cup M)$. To enhance the model’s generalization ability during the replay phase, soft label fusion augmentation is applied to the new constructed dataset. In particular, the Mixup strategy [45] is employed as a form of soft label fusion augmentation. Mixup is used by generating virtual training instances through linearly combining both the sample features and their corresponding labels.

Furthermore, to mitigate the potential label noise issue in historical memory samples during incremental learning, this framework adopts the Mixup strategy for soft label correction. Specifically, new “soft labels” are generated by the weighted fusion of historical memory samples and current new samples. These soft labels are used alongside the original labels during training, allowing the model to not only learn the correct labels of the tasks but also adapt to the inherent fuzziness between labels. This approach helps alleviate error propagation caused by potentially noisy-labeled samples and enhances the model’s robustness.

Given two samples, $x_{B}^{i}$ and $x_{B}^{j}$, in the mixed training batch *B*, with corresponding labels $y_{B}^{i}$ and $y_{B}^{j}$, the new sample generated can be expressed as

(11) $$\begin{array}{cc} x_{i}^{\prime } & =\lambda^{\prime }x_{B}^{i}+\left(1-\lambda^{\prime }\right)x_{B}^{j}\\ y_{i}^{\prime } & =\lambda^{\prime }y_{B}^{i}+\left(1-\lambda^{\prime }\right)y_{B}^{j} \end{array}$$

(12) $$L_{\mathrm{mix}}=-\sum y^{\prime }\log\left(f_{\theta }\left(x^{\prime }\right)\right)$$

Here, λ∼Beta(α,α), and $\lambda^{\prime }=\max\left(\lambda ,1-\lambda \right)$. The synthesized new sample is passed into the model for training alongside the current new sample through data augmentation.

Ultimately, as mentioned in Section 3.1, the objective loss function is enhanced from multiple perspectives of data sample purity and robustness through the above modules. The objective function of the DSCNL method is optimized according to Equation (13):

(13) $$\theta^{*}=\underset{\theta }{\mathrm{argmin}}E_{t∼T}\left\{E_{\left(x_{i},{\bar{y}}_{i},x_{r},{\bar{y}}_{r}\right)∼\left({\bar{D}}_{t}\cup M\right)}\left[L\left(f_{\theta }\left(x_{i}\right),f_{\theta }\left(x_{r}\right),{\bar{y}}_{i},{\bar{y}}_{r}\right)\right]\right\}$$

(14) $$L=L_{\mathrm{ce}}+\beta L_{\mathrm{mix}}$$

where $x_{r}$, ${\bar{y}}_{r}$ represents the clean-labeled sample pairs stored in the memory buffer *M*; $x_{i}$, ${\bar{y}}_{i}$ represents the clean-labeled sample pairs in the current clean-sample subset ${\bar{D}}_{t}$; and $L_{\mathrm{ce}}$ represents the cross-entropy loss function. The hyperparameter β serves to regulate the balance between the components of the loss function.

## 4. Experiments and Results

To comprehensively evaluate the performance of DSCNL in incremental learning under noisy labels, systematic experiments are conducted on two publicly available medical datasets, BloodMNIST and PathMNIST [46] which are both valuable components of the broader MedMNIST collection. The BloodMNIST dataset features blood cell microscope images, comprising 8 distinct classes with 11,959 training samples and 3421 test samples. The PathMNIST dataset, on the other hand, consists of colon pathology images across 9 different classes, containing a larger set of 89,996 training samples and 7180 test samples.Both datasets are standardized to image resolutions of 128 × 128 pixels, and each sample is represented as a (128 × 128 × 3) RGB image.

### 4.1. Experimental Setting

This study follows the widely recognized robust evaluation criteria in the field of incremental learning to ensure the following conditions are met: (1) no overlap in target categories between tasks; (2) all tasks share a unified output space; (3) no task identifiers are required during the inference phase; (4) the experimental setup involves a multi-task scenario, with datasets such as BloodMNIST and PathMNIST divided into multiple incremental tasks.

In terms of noisy label synthesis, a symmetric noise model is adopted. The noise ratio indicates the proportion of noisy labels relative to the entire dataset. The noise level is controlled by setting this ratio and applying a noise transition matrix to perturb the original dataset—specifically, each sample’s true label is randomly changed to any other class in the dataset with uniform probability. Subsequently, the incremental task sequence is constructed by randomly selecting categories, with the specific task category distribution detailed in Table 1. In the class-incremental learning (CIL) setting, sequential class-incremental tasks are defined based on the synthetic noisy label dataset. Specifically, the BloodMNIST dataset is divided into four binary classification tasks: Task 1 (Class 1 vs. Class 2), Task 2 (Class 3 vs. Class 4), Task 3 (Class 5 vs. Class 6), and Task 4 (Class 7 vs. Class 8). Similarly, PathMNIST is split into four tasks, with Tasks 1–3 as binary classification and Task 4 as a three-class classification task. All experiments use ResNet18 as the backbone network, with a total of 50 training epochs.

For the training strategy, the warm-up phase of the auxiliary network is set to 5 epochs. After that, training is adjusted or paused automatically based on data selection needs. The learning rate during the warm-up phase is set to 0.0001, and the batch size is set to 128. A lower learning rate is used to mitigate the risk of model overfitting due to noisy labels, as a high learning rate can cause the model to memorize noisy samples, thus affecting the accuracy of sample selection during optimization and ultimately impairing the model’s generalization ability.

### 4.2. Comparison Methods and Evaluation Metrics

In this experiment, a range of classic incremental learning methods is selected for comparison to evaluate their performance robustness in medical image classification tasks with noisy labels. The comparison methods include the following: (1) Finetune: As a baseline method, the model is directly finetuned on new task data without applying specific incremental learning strategies to mitigate catastrophic forgetting, thus directly influenced by noisy labels. (2) SI [20]: A representative regularization-based incremental learning method. (3) Mainstream experience replay-based incremental learning methods, including ER [43], DER [33], and A-GEM [12].

To quantify the performance of each method, Final Average Accuracy (FAA) is used as the evaluation metric. This metric measures the model’s overall performance by calculating the average accuracy achieved on the test sets of all learned tasks after completing the training of all incremental tasks.

### 4.3. Experimental Results

#### 4.3.1. Experiments on BloodMNIST

**Figure 4:**
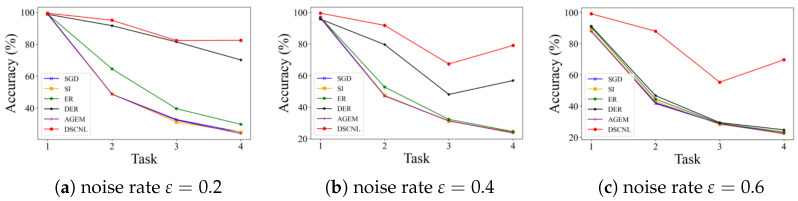
The average accuracy after the completion of each task during training for the comparison methods on the BloodMNIST dataset.

Table 2 and Table 3, and Figure 4, show the experimental results on the BloodMNIST dataset. The results indicate that the proposed DSCNL method achieves optimal performance under all tested noise coefficients, significantly outperforming other methods. Compared to the Finetune baseline, DSCNL achieves an average accuracy improvement of 54.99%, with a maximum gain of 61.21%. In comparison to the second-best method, DER, DSCNL improves accuracy by an average of 24.79%, with a maximum improvement of 41.57%, as shown in Table 2.

The following provides a detailed analysis of each comparison method. Finetune only performs finetuning when learning new tasks sequentially, without any incremental learning mechanism. The results show that as tasks increase, the Finetune model’s classification ability severely degrades, exhibiting significant catastrophic forgetting. Additionally, the performance deterioration is further exacerbated as the noise coefficient increases. SI is unable to effectively maintain its performance in noisy label incremental learning scenarios, as its forgetting mitigation mechanisms are disrupted by noise. A-GEM stores the loss gradients of the samples in the memory buffer. Since noisy labels directly interfere with gradient computation, the performance of A-GEM is significantly affected, leading to poor results. ER uses reservoir sampling to randomly select samples from the training set and store them in the buffer. In a noisy environment, this strategy inevitably stores samples with incorrect labels. In subsequent replay phases, these noisy samples mislead the model’s learning, thereby degrading performance. DER is similar to ER, but it additionally uses Mean Squared Error (MSE) loss to constrain the model’s predicted probabilities for old task samples during the incremental learning process to preserve prior knowledge. However, as the noise coefficient increases, replayed noisy samples still incorrectly influence the model’s predictions, resulting in performance degradation.

Table 3 shows the test accuracy on previously learned classes after completing training for each incremental task. It can be observed that most existing incremental learning methods struggle to effectively handle both the catastrophic forgetting caused by incremental learning itself and the interference introduced by noisy labels. In contrast, the proposed DSCNL method consistently balances the model’s learning plasticity (ability to adapt to new tasks) and knowledge stability (ability to retain old knowledge) across different noise levels. Furthermore, as illustrated in Figure 4, a clear comparison of the average accuracies after each incremental task is presented for different noise rates. This visualization reinforces the DSCNL method’s persistent outperformance under noisy conditions.

#### 4.3.2. Experiments on PathMNIST

**Figure 5:**
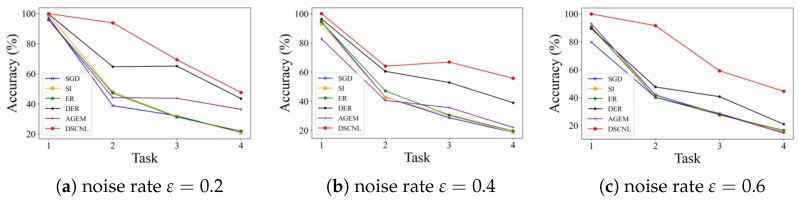
The average accuracy after the completion of each task during training for the comparison methods on the PathMNIST dataset.

Table 4 and Table 5, and Figure 5, present the experimental results on the PathMNIST dataset. Consistent with the observations on BloodMNIST, the proposed DSCNL method demonstrates optimal performance under all tested noise coefficients, significantly outperforming other comparison methods. Compared to the Finetune baseline, DSCNL achieves an average accuracy improvement of 31.02%, with a maximum gain of 43.01%. Relative to the second-best method, DER, DSCNL improves accuracy by an average of 13.19%, with a maximum improvement of 23.64%, as shown in Table 4. The following is a detailed analysis of each comparison method. It is observed that for both Finetune and SI, the negative impact of noisy labels on model performance becomes increasingly severe as the noise coefficient rises. Moreover, the catastrophic forgetting problem remains unresolved under these methods. On the PathMNIST dataset, both ER and A-GEM continue to exhibit suboptimal performance, failing to effectively address the challenges posed by this dataset. DER demonstrates a certain degree of robustness across both datasets (BloodMNIST and PathMNIST). However, as the noise coefficient increases, noisy labels still mislead the model during the replay process, ultimately resulting in performance degradation.

Table 5 presents the test accuracy on previously learned classes after each round of incremental task training. It can be observed that most existing incremental learning methods struggle to effectively address both the catastrophic forgetting caused by sequential learning and the interference introduced by noisy labels. In contrast, the proposed DSCNL method consistently balances model plasticity and stability across different noise levels. Figure 5 visually illustrates the average accuracy on all previously learned task test sets after each incremental task under varying noise rates. This figure further validates the sustained superiority of the DSCNL method in noisy environments.

It is worth noting that, compared to the results on the BloodMNIST dataset, the performance on PathMNIST exhibits certain differences. Therefore, in the following sections, we provide a detailed analysis of the noise filtering mechanism across its two stages and explain its effectiveness.

### 4.4. Effectiveness Verification of the Dual-Stage Sample Selection

To further verify the effectiveness of the proposed dual-stage sample selection strategy, Table 6 and Table 7 quantitatively assess the contributions of the two modules in improving sample purity. Table 6, in conjunction with Figure 5, illustrates the critical role of the auxiliary expert network in enhancing the purity of new task samples during the initial filtering stage. Specifically, Figure 5 shows that the auxiliary network can effectively identify and filter out noisy samples under varying noise levels, thereby significantly reducing the noise ratio in the newly introduced sample subset. As shown in Table 6, the clean-sample subset filtered by the network demonstrates a significant improvement in purity. When converted to equivalent noise ratios of {0.03,0.04,0.06,0.1,0.15,0.23}, this represents an average reduction of 72.77% compared to the original noise ratios, with a maximum reduction of 80%. The network tends to select high-confidence, clean samples, providing a higher-quality data foundation for training the main classification network, which, in turn, improves its classification performance.

Table 7 focuses on the quality of replay samples throughout the incremental learning process, demonstrating that the uncertainty-based sampling strategy is capable of maintaining a high level of sample purity across the entire task sequence. Nevertheless, it is also observed that a small number of noisy-labeled samples inevitably remain in the replay memory, even after filtering. This finding underscores the necessity of implementing robust learning strategies in the subsequent replay enhancement phase to mitigate the potential negative impact these noisy samples may have on model updates.

### 4.5. Comparative Analysis of Replay Sample Selection Strategies

To evaluate the impact of different replay sample sampling strategies on model performance, we conducted a variant study on the DSCNL framework by replacing the original uncertainty-based sampling module with two alternative methods: random sampling and reservoir sampling. Table 8 and Table 9 present the effects of these different strategies on the final test performance and training behavior throughout the multi-stage incremental task sequence.

These results show that, thanks to DSCNL’s effective separation of clean samples in the early stage, all DSCNL variants—regardless of whether they use random, reservoir, or uncertainty-based sampling—can generally obtain high-quality training samples after selection. As a result, they outperform conventional incremental learning baselines in most cases. However, as the noise level in the dataset increases, the performance advantage of the uncertainty-based DSCNL variant becomes more pronounced. This highlights the superiority of uncertainty estimation in selecting informative and high-quality samples to resist the impact of label noise. Table 8 reveals that the random sampling strategy, while simple, fails to maintain class balance, as it purely focuses on sample quantity. This leads to a gradual reduction in the number of samples from earlier tasks, exacerbating catastrophic forgetting. Although reservoir sampling addresses the class imbalance issue by allocating memory slots per class, it still lags behind the uncertainty-based method in terms of performance. The main reason is that reservoir sampling does not consider the representativeness or information value of the samples, whereas the uncertainty-based method prioritizes those most beneficial for learning and knowledge retention.

Therefore, the uncertainty-based replay sampling strategy, by effectively evaluating both sample informativeness and representativeness while preserving class balance, exhibits superior noise robustness and knowledge retention. This capability makes it one of the key factors enabling the DSCNL framework to achieve strong performance in complex incremental learning scenarios.

### 4.6. Impact of Replay Memory Size

Table 10 and Table 11 analyze the impact of different replay memory sizes (specifically set to 200, 500, and 1000) on model performance in noisy incremental learning tasks. Overall, it can be observed that increasing the replay memory size effectively enhances the model’s ability to retain old knowledge, thereby providing stronger resistance to catastrophic forgetting. In most cases, a larger memory buffer corresponds to better preservation of prior knowledge. As the number of tasks increases, the memory allocated to each class gradually decreases, since class-specific replay space is determined by dividing the total buffer size by the number of classes. For example, with a total capacity of 200, the replay space per class in BloodMNIST shrinks from 125 down to 25 as more classes are introduced. Consequently, the model’s learning efficiency for older knowledge diminishes over time. Ideally, the proportion between replay samples and current training samples should remain relatively balanced to maintain stability in learning. However, as part of the overall model design, memory size also brings practical considerations regarding storage cost. Therefore, in practice, it is important to strike a balance between replay buffer capacity and model performance.

### 4.7. Impact of Data Augmentation Strategies

This experiment investigates how different data augmentation strategies used during the replay sample update phase affect uncertainty estimation and, consequently, the effectiveness of diversity-based sample selection. We evaluate the following three augmentation combinations: (1) Affine Trans: affine transformations only. (2) Pixel Trans: pixel-level transformations only. (3) Mix Trans: a combination of affine and pixel-level transformations, additionally incorporating techniques such as CutOut. As shown in Table 12, the use of a mixed augmentation strategy (Mix Trans), which applies a broader range of transformations, more effectively perturbs the samples, enabling a more comprehensive assessment of their uncertainty in feature space. This, in turn, facilitates the selection of samples with higher diversity value for replay. Such a strategy helps the model learn more robust and generalizable feature representations. However, when considering the results in Table 7, a potential trade-off is observed. While uncertainty-based sampling—especially when supported by complex augmentation—significantly enhances replay sample diversity, it also increases the risk of retaining noisy-labeled samples within the clean subset. Specifically, mislabeled samples may appear highly uncertain after augmentation, leading to their unintended inclusion in the replay buffer. This phenomenon highlights the importance of balancing diversity and purity in replay sample selection.

## 5. Discussion

This study precisely targets a highly challenging and practically significant intersection in medical image AI: incremental learning under label noise. In dynamic clinical environments, AI models are required to continuously acquire new knowledge—such as emerging diseases or novel imaging patterns—while existing data often contain varying degrees of labeling errors or inconsistencies. This dual challenge represents a critical bottleneck for the real-world deployment and long-term effectiveness of AI systems in healthcare. This work first identifies a key limitation: existing sample replay-based incremental learning methods suffer significant performance degradation when exposed to noisy labels, rendering them ineffective for complex multi-class classification tasks. This insight is crucial, as the annotation of medical images is both costly and highly susceptible to subjectivity, making label noise the norm rather than the exception. By explicitly integrating noise robustness into an incremental learning framework for medical image analysis, this study addresses this important research gap and establishes a forward-looking research direction with strong practical relevance for building more reliable and adaptive AI systems in real-world medical settings.

Building upon these insights, this study proposes a noise-robust incremental learning framework based on dual-stage clean-sample selection. First, self-filtering in the initial training stage serves as a foundation for high-quality learning. By introducing a clean-sample selection mechanism early in training, the framework reduces the introduction of label noise at the source and lays a solid foundation for subsequent incremental learning and replay. Second, the replay sampling strategy balances both representativeness and diversity. Representativeness ensures that critical information from previously learned knowledge is retained, while diversity—captured through methods such as uncertainty-based sampling (as previously discussed)—allows the model to better generalize and adapt to edge cases. This balanced approach outperforms single-dimensional sampling strategies. Finally, the experience soft-replay mechanism plays a key role in mitigating noise propagation and accumulation. By softly integrating potentially noisy labels during the replay process, it not only enhances the replay data through augmentation but also prevents direct misguidance from erroneous labels, enabling robust learning under label noise. Our framework demonstrates strong performance across multiple simulated class-incremental medical image datasets with noisy labels. In particular, its robustness and generalization advantages are most evident in high-noise and multi-task incremental settings. These results provide compelling evidence for the effectiveness of the proposed framework, showing that it can effectively mitigate catastrophic forgetting while exhibiting strong resilience to label noise.

The effectiveness of our proposed method was rigorously validated through extensive experiments on two medical datasets, BloodMNIST and PathMNIST. As demonstrated in our results, DSCNL consistently achieved optimal performance across both datasets under various noise coefficients, significantly outperforming existing state-of-the-art methods. For instance, on BloodMNIST (Table 2 and Table 3, Figure 4), DSCNL showed an average accuracy improvement of 54.99% over the Finetune baseline and a 24.79% improvement over the second-best method, DER. Similarly, on PathMNIST (Table 4 and Table 5, Figure 5), DSCNL delivered an average accuracy improvement of 31.02% compared to the Finetune baseline. These consistent and substantial performance gaps across both datasets strongly underscore DSCNL’s robust effectiveness and significant advantage in tackling incremental learning challenges in the presence of noisy labels.

Despite the significant progress made in this study toward noise-robust incremental learning for medical image analysis, several avenues remain open for further exploration and improvement:

Firstly, in terms of improving noise separation efficiency and classification performance for complex samples, although the proposed clean-sample selection mechanism performs well overall, a decline in accuracy is observed under high-noise conditions, particularly for classes or samples that are inherently difficult to distinguish in feature space. This suggests that the current approach still has limitations in identifying and separating noisy labels among highly entangled features. Future work should aim to develop more fine-grained noise separation techniques, potentially involving deeper feature analysis or more powerful discriminative models to better handle these “hard” cases and further enhance classification accuracy and robustness.

Secondly, regarding the reuse and value extraction of noisy-labeled samples, the current focus is primarily on filtering and discarding noisy samples. However, the potential utility of identified noisy labels remains underutilized. Future research will explore integrating semi-supervised learning or graph neural networks (GNNs) to automatically or semi-automatically relabel or correct identified noisy samples. This approach could fully leverage all available data, reduce information waste, and further improve generalization and data efficiency.

Thirdly, in exploring rehearsal-free incremental learning, the current framework relies on memory replay to mitigate catastrophic forgetting, which incurs storage overhead and raises potential privacy concerns. Future work will investigate rehearsal-free approaches, particularly those leveraging generative models (e.g., GANs or VAEs), to synthesize representative pseudo-samples as substitutes for real historical data. This would alleviate the need for extensive memory storage, reduce privacy risks, and offer a promising path for deploying incremental learning systems in resource-constrained environments.

In summary, future efforts will focus on improving the precision of noise handling, maximizing data utilization efficiency, and advancing toward more lightweight and adaptive incremental learning paradigms, with the goal of building smarter, more robust, and deployment-ready AI systems for medical image analysis.

## 6. Conclusions

This study delves into a research problem that closely mirrors real-world constraints: the Incremental Noisy Label Problem. This challenge specifically investigates the optimization of models in incremental learning scenarios when confronted with the pervasive issue of noisy labels, a situation particularly prevalent and critical in the field of medical image analysis. By proposing a novel noise-robust incremental learning framework that integrates dual-stage clean-sample selection, a replay sampling mechanism balancing representativeness and diversity, and an experience soft-replay strategy, the proposed approach significantly enhances the model’s continual learning ability, generalization, and robustness under noisy conditions.

The contributions of this work go beyond methodological innovation. Theoretically, it challenges the traditional dependence of incremental learning methods on high-quality labels. Practically, it provides key technical support for developing AI systems capable of operating reliably in real-world medical environments that are inherently dynamic, noisy, and inconsistent in data quality.

While our method demonstrates significant advancements, areas for future exploration are acknowledged. Subsequent work will focus on further optimizing replay strategies and strengthening noise-robust mechanisms to consolidate and extend the achievements of this study. These directions are expected to accelerate the development of smarter and more reliable medical AI systems, paving the way for broader applicability in diverse medical imaging research areas.

## Figures and Tables

**Table 1:** The change in the number of samples for each class under a noise coefficient of 0.3, where B represents the number of samples before noise injection, and A represents the number of samples after noise injection.

		Class 1	Class 2	Class 3	Class 4	Class 5	Class 6	Class 7	Class 8	Class 9
BLOODMNIST	B	852	2181	1085	2026	849	993	2330	1643	-
	A	1073	1956	1239	1843	1074	1140	2023	1611	-
PathMNIST	B	9366	9509	10,360	10,401	8006	12,182	7886	9401	12,885
	A	9601	9496	10,196	10,380	8637	11,479	8592	9698	11,917

**Table 2:** The Final Average Accuracy (FAA) values of each method on the BloodMNIST dataset under different noise coefficients.

Methods	Noise Rates					
	0.1	0.2	0.3	0.4	0.5	0.6
Finetune	24.95	24.86	24.55	24.2	23.54	22.88
SI	25	24.82	24.77	24.31	23.74	22.88
ER	37.41	29.75	25.02	24.49	23.48	23.39
DER	81.59	70.19	56.24	56.81	33.24	28.15
AGEM	24.88	24.03	24.69	23.59	23.56	22.33
DSCNL	86.16	82.47	84.02	79.09	73.51	69.72

**Table 3:** The test accuracy of each method on the BloodMNIST dataset after the completion of each task under a noise rate of 0.3.

Methods	Task Query			
	Task 1	Task 2	Task 3	Task 4
Finetune	97.35	-	-	-
	0	93.93	-	-
	0	0	95.64	-
	0	0	0	98.24
SI	98.73	-	-	-
	0	94.61	-	-
	0	0	95.26	-
	0	0	0	99.12
ER	97.7	-	-	-
	26.15	96.74	-	-
	0.46	1.01	95.83	-
	0.23	0.11	0.57	99.21
DER	98.04	-	-	-
	61.75	95.39	-	-
	64.17	73.37	87.29	-
	44.12	65.17	29.79	85.92
AGEM	98.27	-	-	-
	0.00	87.19	-	-
	0.00	0.00	96.77	-
	0.00	0.00	0.00	98.77
DSCNL	99.08			
	94.59	91.91		
	90.78	50.11	96.77	
	93.55	50.57	92.22	99.65

**Table 4:** The Final Average Accuracy (FAA) values of each method on the PathMNIST dataset under different noise coefficients.

Methods	Noise Rates					
	0.1	0.2	0.3	0.4	0.5	0.6
Finetune	21.83	20.99	20.72	18.66	17.77	15.24
SI	21.99	20.74	20.98	18.68	17.3	15.55
ER	24.93	21.91	20.62	19.61	16.48	16.41
DER	54.21	43.49	41.09	38.96	23.62	20.83
AGEM	35.83	36.34	32.96	22.03	19.02	14.44
DSCNL	56.99	47.56	63.73	55.71	32.88	44.47

**Table 5:** The test accuracy of each method on the PathMNIST dataset after the completion of each task under a noise rate of 0.3.

Method	Task Query			
	Task 1	Task 2	Task 3	Task 4
Finetune	97.76	-	-	-
	0	82.22	-	-
	0	0	94.59	-
	0	0	0	82.88
SI	94.46	-	-	-
	0	84.79	-	-
	0	0	90.23	-
	0	0	0	83.92
ER	97.66	-	-	-
	1.98	77.34	-	-
	0.6	0.78	90.49	-
	0	0	0	82.51
DER	98.81	-	-	-
	41.81	95.09	-	-
	20.82	63.73	89.84	-
	13.05	51.34	17.71	82.29
AGEM	97.93	-	-	-
	96.51	0	-	-
	2.48	21.32	88.41	-
	4.64	25.45	18.88	82.90
DSCNL	99.95			
	97.84	97.32		
	63.79	77.46	93.03	
	72.70	73.10	18.82	90.32

**Table 6:** The clean-sample selection rate of the auxiliary expert module at each task stage on the BloodMNIST dataset.

Noise Rate	Task Query				
	Task 1	Task 2	Task 3	Task 4	Mean Purity
0.1	97.57	95.65	92.82	99.11	96.28
0.2	97.67	94.29	89.94	98.62	95.13
0.3	95.66	91.31	87.02	98.95	93.23
0.4	94.74	87.57	77.87	98.22	89.6
0.5	88.88	85.64	68.31	96.02	84.71
0.6	82.66	75.07	54.32	95.17	76.8

**Table 7:** The clean-sample retention rate of the uncertainty-based sampling module at each task stage on the BloodMNIST dataset under a noise rate of 0.3.

Task Query	Class								
	Class 1	Class 2	Class 3	Class 4	Class 5	Class 6	Class 7	Class 8	Mean Purity
Task 1	98.0	96.8	-	-	-	-	-	-	97.4
Task 2	96.8	98.4	94.4	94.4	-	-	-	-	96
Task 3	96.42	100	92.85	94.04	94.04	82.5	-	-	93.3
Task 4	95.23	100	90.47	93.65	92.06	85.71	100	100	94.64

**Table 8:** The Final Average Accuracy (FAA) on the BloodMNIST dataset under different sampling strategies.

Methods	Noise Rate					
	0.1	0.2	0.3	0.4	0.5	0.6
DER	81.59	70.19	56.24	56.81	33.24	28.15
DSCNL (w.random)	62.65	56.84	64.64	61.5	50.25	48.87
DSCNL (w.res)	83.12	79.15	79.56	82.09	68.85	66.39
DSCNL (w.uncer)	84.73	82.47	84.02	79.09	73.51	69.72

**Table 9:** The test accuracy on the BloodMNIST dataset under a noise rate of 0.3 for models using different sampling strategies.

Methods	Task Query			
	Task 1	Task 2	Task 3	Task 4
DER	98.04	-	-	-
	61.75	95.39	-	-
	64.17	73.37	87.29	-
	44.12	65.17	29.79	85.92
DSCNL (w.random)	98.39			
	92.97	84.04		
	56.34	49.66	97.15	
	30.88	31.46	96.58	99.65
DSCNL (w.res)	96.08	-	-	-
	97.47	88.88	-	-
	85.60	47.87	96.96	-
	87.44	46.07	85.20	99.56
DSCNL (w.uncer)	99.08			
	94.59	91.91		
	90.78	50.11	96.77	
	93.55	50.57	92.22	99.65

**Table 10:** The Final Average Accuracy (FAA) of the model on the BloodMNIST dataset under different sampling strategies.

Buffer Size	Noise Rate					
	0.1	0.2	0.3	0.4	0.5	0.6
200	76.86	67.93	68.51	68.79	64.17	46.50
500	84.73	82.47	84.02	79.09	73.51	69.72
1000	87.77	85.98	86.23	84.35	78.79	79.15

**Table 11:** The Final Average Accuracy (FAA) of the model on the PathMNIST dataset under different replay memory sizes.

Buffer Size	Noise Rate					
	0.1	0.2	0.3	0.4	0.5	0.6
200	56.29	37.41	37.46	33.91	30.72	24.13
500	56.99	47.56	63.73	55.71	33.80	43.21
1000	63.89	67.64	59.65	59.55	47.23	45.98

**Table 12:** The Final Average Accuracy (FAA) of the model on the BloodMNIST dataset under different data augmentation combinations.

Trans Type	Noise Rate					
	0.1	0.2	0.3	0.4	0.5	0.6
Affine Trans	85.69	76.86	78.96	76.52	71.54	65.93
Pixel Trans	84.77	80.30	80.57	74.68	63.77	54.71
Mix Trans	86.16	82.47	84.02	79.09	73.51	69.72

## Data Availability

No new data were created or analyzed in this study.
